# Global public health today: connecting the dots

**DOI:** 10.3402/gha.v9.28772

**Published:** 2016-02-18

**Authors:** Marta Lomazzi, Christopher Jenkins, Bettina Borisch

**Affiliations:** 1 World Federation of Public Health Associations, c/o Campus Biotech, University of Geneva, Geneva, Switzerland; 2 Institute of Global Health, c/o Campus Biotech, University of Geneva, Geneva, Switzerland

**Keywords:** global health today, politics, global health education, global public health leaders

## Abstract

**Background:**

Global public health today faces new challenges and is impacted by a range of actors from within and outside state boundaries. The diversity of the actors involved has created challenges and a complex environment that requires a new context-tailored global approach. The World Federation of Public Health Associations has embarked on a collaborative consultation with the World Health Organization to encourage a debate on how to adapt public health to its future role in global health.

**Design:**

A qualitative study was undertaken. High-level stakeholders from leading universities, multilateral organizations, and other institutions worldwide participated in the study. Inductive content analyses were performed.

**Results:**

Stakeholders underscored that global public health today should tackle the political, commercial, economic, social, and environmental determinants of health and social inequalities. A multisectoral and holistic approach should be guaranteed, engaging public health in broad dialogues and a concerted decision-making process. The connection between neoliberal ideology and public health reforms should be taken into account. The WHO must show leadership and play a supervising and technical role. More and better data are required across many programmatic areas of public health. Resources should be allocated in a sustainable and accountable way. Public health professionals need new skills that should be provided by a collaborative global education system. A common framework context-tailored to influence governments has been evaluated as useful.

**Conclusions:**

The study highlighted some of the main public health challenges currently under debate in the global arena, providing interesting ideas. A more inclusive integrated vision of global health in its complexity, shared and advocated for by all stakeholders involved in decision-making processes, is crucial. This vision represents the first step in innovating public health at the global level and should lead to a serious rethinking of education curricula to allow the next generation to engage within political contexts for restructuring global public health.

## Introduction

The integration and interactions between numerous cultural, economic, social, environmental, governmental, and political processes are characteristic of our complex globalized world. These processes exist at all levels, from global to local (1, 2).

Global public health is conceptualized by the recognition that the processes that underpin globalization have led to an increased vulnerability of public health. Impacted by a wide range of actors, both from within and without boundaries, global public health faces new challenges (3).

Health and politics interact in increasingly connected and multidirectional ways. The connection between public health and other political aspirations, such as economic development, equity, and stability, is more explicitly being recognized (4). Equally it is increasingly understood that health is a product of numerous dynamics at different levels of governance; it is often determined by complex political processes dealing with economic, commercial and foreign affairs issues (5, 6) and impacted by social, environmental, and behavioral health determinants, including economic constraints, demographic changes, unhealthy lifestyles, and living conditions (7).

The diversity and proliferation of the actors involved creates challenges for global public health and has led to an environment that is too complex for a business-as-usual approach (8). Good health outcomes cannot be delivered by national health systems alone. Multidimensional challenges need substantial, comprehensive, diverse, and creative responses (9). Governance systems that are better structured and financed and adapted to operate effectively within multilateral systems are required to complement issue-based initiatives (10). Effective responses are needed to engage with sectors outside of health, such as commercial organizations and the private sector (10). Philanthrocapitalism and public health programs relying on public-health partnerships (PPPs) now characterize much of the public health agenda (11, 12). With an increased international focus on issues related to health (i.e. G7, G20, WEForum, and the COP21 (13, 14)), the reflections in this paper are increasingly relevant.

Several aspects of global public health today need debate and discussion (15). These questions include, who shall lead global public health today? What skills are required by public health professionals? What are the fields of action? How can coordination and concerted decision-making processes be implemented at the global level? How can the political environment be better engaged and should there be better frameworks around negotiation for public health issues?

As per the request of Dr M. Chan, director-general of the World Health Organization (WHO), the World Federation of Public Health Associations (WFPHA) (16) has embarked on a collaborative consultation with the WHO to investigate how to adapt public health to its future role in global health. High-level stakeholders worldwide have engaged in this discussion.

This paper aims to highlight some of the outcomes from this consultation and to attempt to better place the global public health community for tackling the contemporary and complex health problems in our globalized world.

## Methods

The study started in 2013. A teleconference was organized with selected public health stakeholders worldwide to reflect on the need to renew the meaning of public health in relation to the changing global context. The group also sought to identify terms of reference for a concept note to be discussed with the director-general of the WHO. In parallel, the WFPHA conducted a literature review on global public health (17).

The teleconference was followed by a qualitative study (see Annex I). Questions were formed by a core group (WFPHA and WHO) and pretested by a small group of participants. The questions dealt with the most important challenges for public health today, the definition of leadership in public health, and skills required by public health professionals, as well as the fields of actions for public health. The development of global public health in terms of short, middle, and long-term objectives was debated. Data collection was performed by email between February and April 2014.

Inductive content analysis was conducted (18). First, each answer was reviewed line by line and coded according to the main subject categories. Subsequently, similar categories were grouped into themes (*main public health challenges and needs*, *leadership in public health*, *(new) public health professional skills*, *fields of action*, and *global health framework*; see Annex II). The different themes are described in the Results section, reporting as examples the most relevant statements (shown in the tables) or summarizing the main outputs and categories of selected themes (shown in the figures), to draw attention to the main points.

A description of the analysis and presentation of the results follow the recommendations given in the ‘Guidelines for Critical Review Form: Qualitative Studies’; however, the researcher's bias and influence of the researcher's point of view cannot be completely avoided as per the methodology itself (19).

The 24 stakeholders who took part in the consultations were well respected within the field of global public health, holding important positions such as dean or executive officer in leading universities, institutes, non-government organizations (NGOs), multilateral/bilateral organizations, governments, and so on from different public health–related fields (public health in general, health education, health economy, environmental health, and urban health, to cite a few) and covering all WHO regions. Moreover they represented different categories of public health–related professionals (medical doctors, nurses, master in public health (MPH), architects, health economists, health journalists, etc.).

## Results

### Main public health challenges and needs

Interviewees described most of the public health needs and challenges currently under debate in the global arena (Tables 1 and 2; topics are reported in order of importance in descending order).

**Table 1:** Main needs in global public health (themes and citations)

Main needs	Supporting Quotes
Government engagement and understanding of public health	‘… health care decision makers and political leaders do not know or understand the scope of services of public health and its role in combating diseases and wellbeing of the population individually and at the community level’.
Multisectoral, holistic, and inclusive approach based on preventive healthcare	‘An environment that is inclusive, transparent and evidence-based is the only possible way to address multi-faceted and complex challenges … The old paradigm and stereotyped roles (often countering each other) of public, private and civil society organizations will not be conducive to dialogue, and constructive innovative ideas, and belongs to a medieval view of the world’.
Global or at least regional cooperation of advocacy, education, and research	‘The UN framework is much too slow in decision making … we need the intermediate step of regional binding cooperation, a common language (EPHF), a close cooperation of advocacy, education and research (in Europe EPHA, ASPHER and EUPHA), and regular well-funded meetings of public health professionals and advocates’.
Sustainable and accountable funding of infrastructure, human and organizational resources, and technologies and services	‘International trade deals pose serious threats to public health. The creation of investor trade dispute mechanisms mean that corporations can constrain government attempts to protect public health’.
Appropriate education and training as a base to guarantee effective interventions	‘[One of the challenges is] completely ignoring the training of and capacity building of public health professionals, assigning it to national governments and expecting effective implementation of interventions’.

ASPHER = Association of Schools of Public Health in the European Region; EPHF=Essential Public Health Functions; EPHA=European Public Health Alliance; EUPHA=European Public Health Association.

**Table 2:** Main challenges in global public health (themes and citations)

Main challenges	Supporting Quotes
Commercial determinants of health, corporations’ power, neoliberal ideology and economic liberalism	‘International trade deals pose serious threats to public health … A few large corporations (e.g. News Corp) have the power to shape the global discourse’
Social inequalities and health within the society and between countries	‘The particular challenge is the social gradient in health … we should not only be addressing poverty and health, but social inequalities and health’
Climate change catastrophe, global environmental changes, and planetary destruction • Limited understanding on how climate change impacts upon people's health by politicians and professionals • Absence of systematic quantification of climate change's impact on health	‘The basic building blocks of health i.e. a safe healthy immediate living environment have been forgotten’
Old, partial, and often not reliable data and indicators in many programmatic areas Lack of investment in data collection	‘Most data in the global burden of disease are estimates! … The average supermarket chain in the west has far more information about individuals than many public health departments’ ‘No one invests in data collection, how they can generate good data? … No it is the biggest tragedy in public health’

### Leadership in public health

Some of the interviewees agreed with the idea that there is a wide agreement to view public health beyond the health care sector and that thus a ‘whole of government approach’ is required, while the rest of the interviewees underscored that public health is often marginalized by governments and that the Health in All Policies (HiAP) approach (20) has not been implemented.

Many governments marginalize public health. With a very few exceptions (e.g. Jens Stoltenberg, former Norwegian PM) most leading politicians pay lip device to public health. Almost always trade trumps health

The application of the HiAP approach embraces inclusive decision-making processes, formulation of effective policies to achieve measurable and sustainable goals (with a special focus on tackling inequalities), transparency, accountability, and support for research and innovation. Politicians should guarantee that health is integrated into all policies, focusing on the health of the population and not merely on economic prosperity and perceived health in its economic dimension (health equals wealth). The role of the private sector has been acknowledged, but its participation should be regulated to limit the influence of commercial interests on policy setting. The WHO should provide leadership and play a supervisory and technical role.

Public health will gain greater mileage when the politicians assume leadership of the systems approach and that is borne [out] by history. Great achievements in public health in the past were not driven by public health professionals … Public health should be driven by politicians and I hope soon leadership of countries will not only be based on economic prosperity for the citizens but the healthy living for the population

In this context, leadership in public health should reflect the penetration of public health into all sectors of the government and society. It should be based on the work of accountable champions, not necessary from the health arena, that understand the different sectors and have an integrated vision of the leverages and available resources to achieve common goals.

Public health has to be liberated from the medical agenda and become truly interdisciplinary and multiprofessional.

The immediate, midterm, and longer-term implications of the intersectoral collaboration should be understood; the sophistication of working across sectors increased; and innovative programs put in place, monitored, and evaluated.

The ability to ‘connect the dots’ is increasingly a key component of leadership … Lack of leadership is visible when only the voice of few is heard …

Public health professionals need to engage in the big dialogues: sustainability, social protection, development, climate change, poverty, inequality, governance, trade, intellectual property, migration, food, and climate change, to cite a few.

Drill down from megatrends: what are the immediate, midterm and longer term implications for PH and their respective inputs of other sectors? Are there inter-linked impacts on both health and the other sector that justify a joint solution? Understand and use impact analysis (to note there are over 40 methods) and measurement of health inequalities that are based on determinants of health approaches. Use the data to discuss possible mutually beneficial goals

Leadership should be able to communicate with government and society that public health is a sustainable, cost-effective means to better living even in complex environments and should be able to build a social movement for public health.

Links with civil society and to specific groups, such as Transparency International and Human Rights Watch, adopting a lobbying approach, should be better exploited.

Leadership should be visionary and lead with credibility, respect, and courage.

### (New) public health professional skills

**Fig. 1:**
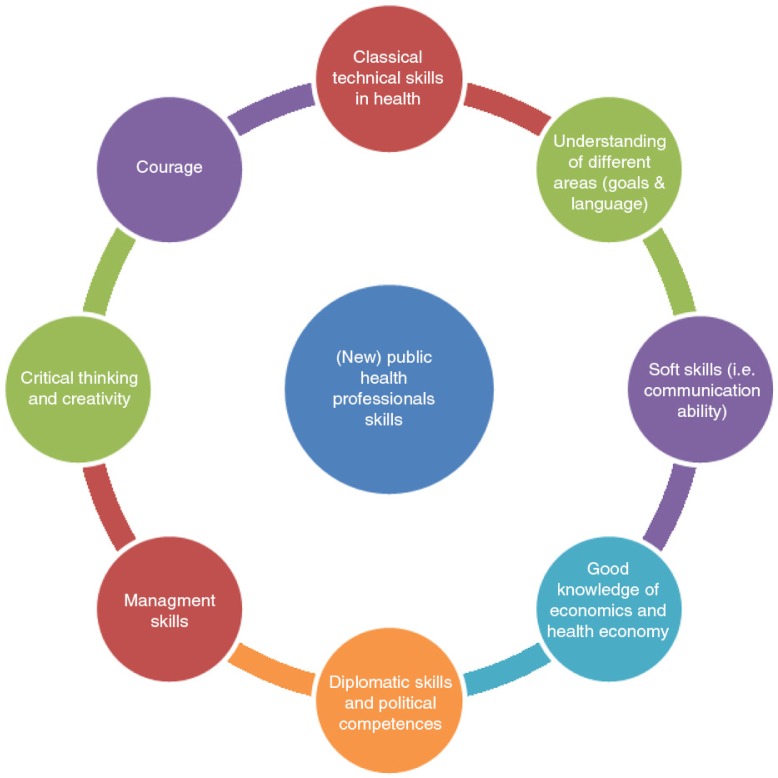
(New) public health professionals’ skills.

Health workers are a public good and their training should be tuned to the current social-health reality and adequate to the world of work where they will act. Numerous skills are necessary and could be provided in part through an appropriate master's program (Fig. 1) and intersectoral education (such as standalone academic faculties of public health/health sciences or schools of government in health). Skills harmonization at least at the regional level has been recommended.

### Fields of action

**Fig. 2:**
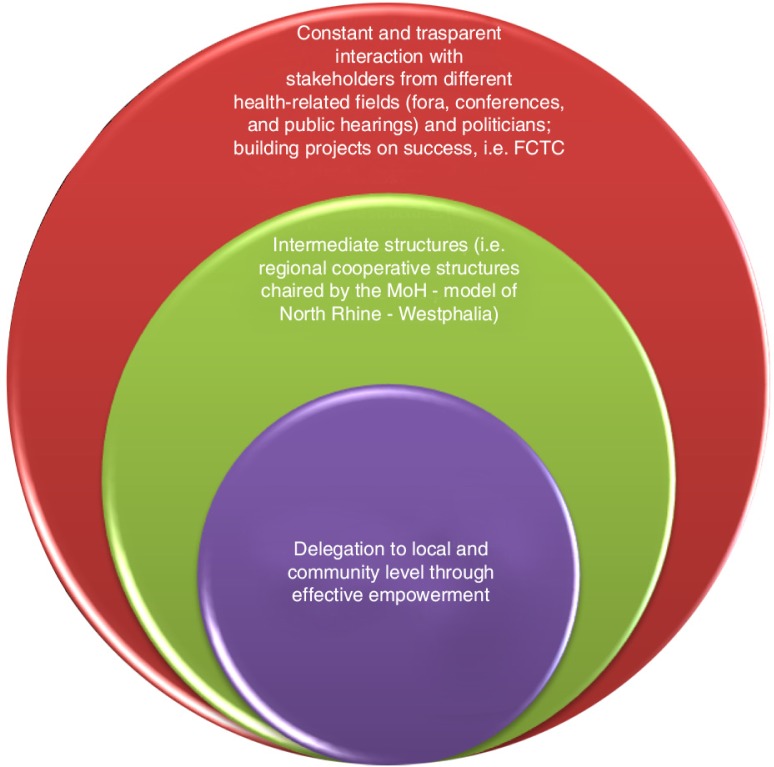
Roles and fields of action of public health professionals.

Stakeholders underscore that public health professionals should cover different roles at different levels. They are key in the concerted decision-making process; this process should be implemented with closer and more transparent interaction with authorities and non-state actors to reach targets in a realistic time frame, taking advantage of political windows of opportunity and creating strong links with communities (Fig. 2).

Public health people can, and should, fill a variety of roles. Some will be involved in research and monitoring, others will be involved in policy development, others in implementation. Sometimes these skills may all be embodied in one person, but they need not be

The adoption of a public health impact assessment (21) analogous to environmental impact assessments (22) has been suggested to guarantee compulsory consultation of public health professionals.

### Global health framework

Some of the interviewees supported the idea of a framework of instruments or of common structures for negotiation and collaboration to influence governments. The framework should be specifically tailored to the circumstances, needs, and cultures of each country. The agreement under the framework should be respected by politicians, although its non-mandatory nature and efficiency should be measured.

Frameworks are great for dialogue and will be a useful tool. What is lacking is convincing the politicians about the need for the frameworks and to respect the agreements reached under the frameworks. Most of the frameworks I have seen tend to neglect the need to measure overall assumptions that the interventions will work

Several principles have been suggested for the framework with the idea that the principles should be multipliers. The need for political skills, imagination, initiative, and courage to face the different issues cannot be ignored.

Other interviewees highlighted multifaceted proposals to build consensus among all interlocutors at the national and international levels as well as with the WHO.

Interlocution with governments which would include academic-institutional leaderships, representatives of scientific societies, leaders of organized social movements interested in health, parliamentarians, associative instances of health managers (in Brazil we have the Municipal and State Heath Secretaries Councils) and international health representations (Pan American Health Organization (PAHO), WHO)

WHO and the public health community need to coalesce and synthesize the various jargons: social determinants for health, environmental and other determinants for health, Health in All Policies, intersectoral action for health, multi-sectoral action for health, and so on

## Discussion

The consultation aimed to provoke a valuable discussion on how to adapt public health to its new role in the global context with a special focus on the next generation of global public health leaders.

In this section we discuss the main ‘controversial’ outputs.

Today public health professionals face a number of profound and complex challenges. The recognition of multiple determinants of health, from the socioeconomic to environmental, across multiple levels, from the national to individual levels, is important.

Solutions should be prioritized that involve different sectors and actors, such as multidisciplinary researchers from different academic backgrounds, institutional decision-makers, representatives of civil society, and the private sector (23).

New skills are required to negotiate the interface between varied groups with different interests, legitimacy, and power. Creativity and commitment should underpin the new generation of global public health professionals willing to take the initiative to lead global changes for the next decades (24).

Important changes are necessary to prepare the public health workforce for its future role. First, the public health profession itself has not yet been acknowledged nor legitimized. The public health workforce consists of different professionals; efforts to shape and legalize this profession so that it can be part of the elite of regulated professions are made at the regional level (25).

Second, public health education has not yet recognized that the socioeconomic, political, environmental, and commercial changes impacting public health require specific and adapted teaching and training to address these contexts. Meeting these challenges implies profound changes in terms of education and training. An interesting example is provided by the pioneering experience of Brazil, in their Health Government Schools program, intra- and intergovernmental training of professionals that aims to provide the skills necessary to tackle public health challenges with a multisectoral approach (26).

Higher academic institutions are searching for appropriate strategies in competence-based education, which will increase the global attractiveness of their academic programs and courses for continuous professional development. Such educational reform will be a long and difficult process that demands leadership and requires effort in changing perspectives and work styles and creating good relationships between all potential stakeholders (27). New developments in public health training, including flexible academic programs and lifespan learning with a focus on employability and accreditation, are key to better preparing future professionals in global public health (28). Moreover, since public health faces more and more global opportunities and challenges, higher education institutions worldwide have to look beyond national boundaries and participate in networks for education, training, research, development, and practice (29).

Concrete ideas about how to equip the new generation of public health professionals with the necessary political sensitivity and awareness, as well as a serious rethinking of the education curriculum, are necessary. The new curriculum should cover several ‘global’ issues such as health politics and security beyond borders; social, commercial, and economic determinants of health; and humanitarian issues. It should provide the skills to understand and interact with the commercial industry as well as to address lifestyles and behaviors, to cite a few. The curriculum should be harmonized; although such harmonization does not currently exist at the global level, some activities are ongoing at the regional level. Examples include the Association of Schools of Public Health in the European Region and partners (23, 30) and the South American Countries Union through the South American Institute of Government in Health (31). However, the set-up of a global collaborative approach to public health education in a short time frame remains crucial (32).

Updated and reliable data are not always available, especially in certain programmatic areas of public health or in developing countries. This point was largely debated during the consultation. Over the past decades, companies, in particular pharmaceutical and insurance companies, have collected data from research and development programs, patients’ information, clinical trials, and so on, creating ‘big data’ databases. Improved statistical and computational methods have allowed optimized use of the data, and enhanced health care applications have been developed (33). Despite the potential positive effects on public health, for the most part only multinational health care companies have had the access and the resources to exploit these data sets (34). Several criticisms have been raised, underscoring the risk that big data will not be used as a public good, but only for informing commercial decisions. (35). In this context, effective and accountable private–private partnership are of primary importance.

Health is a political choice, and it is always made within the context of competing interests (36). The need for genuine political engagement has been stressed in this consultation. Looking at health through the lens of political determinants means analyzing how different power dynamics, institutions, processes, interests, and ideological positions affect health within different political systems and cultures and at different levels of governance. Politicization of health occurs at all levels of governance, and in an increasingly globalized context it occurs within governments, between governments, within global institutions, and in the private sector and civil society. Health has become an integral feature within processes of globalization related to trade, commerce, foreign policy, and security (37), while gaining greater prominence in national affairs due to the increased recognition of the economic rationale for investing in health.

Philanthropic and business interests are deeply linked to global public health. There are now dozens of big global health PPPs with budgets ranging from a few million to billions of dollars, such as Stop TB, Roll Back Malaria, the International AIDS Vaccine Initiative, and the Global Alliance for Improved Nutrition, many of which are supported by the Bill and Melinda Gates Foundation (BMGF). Others such as the Global Fund or Gavi are shaping, as with BMGF, global public health through the PPP model. The work of the WHO has also become more and more interconnected with the activities of PPPs, merging the commercial mandates of industries with the WHO's engagement with health as a human right. These partnerships shape the current and future global public health scenario, making commercial interests compromise with the public health agenda and providing legitimacy to corporations’ activities through association with UN agencies. Despite the undeniable conflict of interest, only this kind of partnership currently has the financial and human resources to sustain effective global health interventions (11).

In this context, a compulsory consultation of public health professionals for certain policies should be guaranteed. Recognizing that health has always been political in nature, there is now the need for a clear affirmation that the lack of engagement with the field of public health in political processes needs to change. This is important for ensuring that health outcomes are considered across all policy decisions, both nationally and beyond national jurisdictions. The adoption of a public health impact assessment is an important example that improves accountability in relation to political decisions impacting health. Even if this assessment is not a new instrument, it is a crucial one for improving population health, since it calls for different sectors to work together, combines several issues of relevance, and synthesizes them for purposes of decision making (21).

However, public health remains marginalized by many governments (38). Challenging this marginalization needs to be a central focus of public health professionals. Rhetoric, not action, has characterized approaches to health, with priorities often being given to economic and commercial interests. The Lancet and the University of Oslo's Commission on Global Governance for Health have highlighted the central importance of political determinants on health, analyzing the political origins of health inequity as well as the power disparities and dynamics across a range of policy sectors defining the global political determinants of health as the product of the global political interaction across all sectors that affect health (39). Politics, both nationally and internationally, throughout and prior to the recent Ebola outbreak in West Africa provide further illustration of how political determinants shape outbreaks and outbreak responses (40, 41).

Through this study, a number of milestones in public health that could be used as examples as well as innovative solutions have been highlighted to put public health in the global spotlight. Such milestones include building on successes like the Framework Convention on Tobacco Control (42), or participating in fora allowing continuous and constructive discussions with stakeholders from different fields, as well as taking advantage of intermediate regional or international structures. Examples include the regular health conferences sponsored by the WHO Healthy Regions Program, between all relevant stakeholders and chaired by ministers of health, based on the North Rhine-Westphalia model (43), and the Brazilian Municipal and State Health Secretaries Councils, which gather academic institutional leaders, representatives of scientific societies, leaders of organized social movements interested in health, parliamentarians, and top-level health managers, as well as international health representatives from the WHO or PAHO (44).

Although very interesting reflections and examples have been provided, interviewees have not always proposed groundbreaking solutions that encompass a comprehensive and intersectoral vision of global health in its broader and complex world context, instead focusing mainly on their areas of expertise. Public health today needs a new integrated vision of the different dots with an integrated analysis of the leverage points and available resources to achieve common goals and to add public health input into political decision-making processes.

Many questions remain to be addressed. On the one hand, the question of whether today we need public health professionals trained to work with other sectors or whether we should provide basic training in public health to any professional is still under debate. How can public health schools guarantee appropriate education and training? Is the model of an independent school of public health with an updated program the solution? Or would we do better to have courses of public health within all other faculties and governments? Shall we focus education on pre- or post-graduate students?

On the other hand, the global public health leadership needs to respond to today's complex context. Who should lead global public health in the future? What is the role of governments, multilateral/bilateral organizations, and NGOs? How do we engage multinational companies and philanthrocapitalism for global public health?

Important frameworks have and will characterize the story and progress of global public health. Over the last 15 years the Millennium Development Goals (MDGs) have represented a mission and a movement to target public health efforts and investments, leading to important achievements. Beyond the MDGs, in-depth discussions and consultations have been organized to define the development framework after 2015 (45). At the Sustainable Development Summit, UN member states adopted the 2030 Agenda for Sustainable Development, which includes a set of 17 Sustainable Development Goals to eliminate poverty, combat inequality and injustice, and tackle climate change (46). At the global level, the WFPHA together with the WHO has embarked on an initiative to stimulate a debate around the global public health concept and the creation of a flexible framework for advocacy. The framework, called “A Global Charter for the Public's Health” (unpublished observation), can be applied globally in different countries and settings and provide a common set of recommendations. The common conceptualization of global public health is required to inform future professional, organizational, and political actions, whereas the ‘charter’ for sustainable and secure health infrastructures and services is essential to support health in everyday life and to minimize the negative economic, social, and environmental impacts of globalization on the health of all populations.

## Conclusions

The study highlights some of the main public health challenges currently under debate in the global arena, such as the need to prepare the public health workforce for its future role, to have updated and reliable data, and to ensure governments’ engagement towards a HiAP, accountable, and multisectoral approach.

Remarkable suggestions and models from countries where public health has a strong voice and is integrated into government policies and initiatives should be carefully taken into consideration.

However, we feel that a more comprehensive and integrated vision of global health in its complexity, shared among all stakeholders involved in decision-making processes, is still missing. This shared vision of global public health represents the first step in innovating public health at the global level and should lead to a serious rethinking of education curricula to allow the next generation to engage with political contexts for structuring public health action. Global leaders are essential to realize this change, with the final aim to provide a healthy, sustainable, and stable society.

## Annex I

Survey questions:

1. Given the important changes and developments over the last 15 years, where do you see the most important challenges for public health today?
2. As there is a wide agreement to view public health beyond the health care sector and that thus a ‘whole of government approach’ is required, how would you define leadership in public health?
3. In light of increasingly complex health negotiations and in order to address the determinants of health, which skills are required by (new) public health professionals?
4. As there is agreement that in many programmatic areas of public health there are good data available, which should be the fields of action of public health?
5. If we want to safeguard health in the global arena, how can we act more proactively and how can we better understand the agendas in other sectors?
6. How can the concerted decision-making process be implemented? How far should public health people be involved in the implementation of the agreed-upon policies? How to address the challenges of the rapidly changing environment, the administrative imperatives and leverages?
7. In light of the significant advocacy role that public health professionals play, do you think that a common framework of instruments to influence governments would allow more effective negotiations? Which set of principles should be included in the framework?

## Annex II

**Table:**

Theme	Categories (in alphabetical order)
Main public health challenges and needs	• Global governance and cooperation
	• Financing
	• Climate and environmental change and urbanization
	• Inequalities
	• Public health workforce and infrastructures
	• Data availability
Leadership in public health	Leadership in public health should (when agreed – most cited):
	• Be multisectoral, multidisciplinary, and all-inclusive
	• Have concrete terms, goals, and approaches
	• Advocate for public health with governments and civil society
	• Assure sustainable financial resources to health
	• Be innovative, visionary, and accountable
(New) public health professional skills	• Leadership skills
	• Technical skills
	• Multisectoral knowledge and understanding
	• Soft skills
	• Financial skills
	• Diplomatic and advocacy skills
	• Management & analytical skills
	• Other skills (such as accountability, creativity, open-mindedness, and courage)
Fields of action	• Interaction with state and non-state actors and communities
	• Policy development and implementation
	• Research and monitoring
Global health framework	• Be tailored
	• Be respected by governments
	• Be evaluated
	Set of principles (when agreed – most cited):
	• Multiplier principles
	• Economic saving and sustainable financing
	• Intersectoral dialogue and collaboration
	• Policies and advocacy
	• Public health education
	• Social determinants of health
	• Transparency, equity, and ethical conduct
